# Innate immune overactivation hinders nuclear reprogramming through IFN-IFNAR1 axis

**DOI:** 10.1016/j.bbrep.2025.102395

**Published:** 2025-12-03

**Authors:** Zhimin Song, Yaofeng Wang, Yun Zhang, Jingjing Chen, Tinghong Zhang, Shu Meng

**Affiliations:** a State Key Laboratory of Respiratory Disease, The First Affiliated Hospital, Guangzhou Medical University, Guangzhou, Guangdong, 510120, China; b Zhongshan School of Medicine, Sun Yat-sen University, Guangzhou, Guangdong, 510080, China; c Department of Basic Science Research, Guangzhou National Laboratory, Guangzhou, Guangdong, 510005, China

**Keywords:** Innate immunity, Nuclear reprogramming, IFN-IFNAR1 axis

## Abstract

Nuclear reprogramming to pluripotency holds great promise for regenerative medicine. However, innate immune overactivation may impair nuclear reprogramming efficiency but the underlying mechanism is not fully understood. Here we used mouse embryonic fibroblasts isolated from doxycycline-inducible OSKM transgenic mice as the nuclear reprogramming system and administered a high dosage of Polyinosinic polycytidylic acid (PIC) to stimulate TLR3 as the innate immune overactivation. PIC treatment reduced nuclear reprogramming efficiency. We identified that PIC treatment upregulated type I interferon transcription and secretion, IFNAR1 cell surface expression, and nuclear Stat1 level. Importantly, the introduction of Ifnar1 blocking antibody completely reversed this impaired nuclear reprogramming efficiency. Similarly, Ifn-β neutralizing antibody substantially ameliorated the impaired nuclear reprogramming efficiency caused by PIC treatment. Our data suggest that innate immune overactivation impairs nuclear reprogramming through the IFN-IFNAR1 axis. Blocking this signaling pathway can be used as a general strategy to enhance nuclear reprogramming efficiency.

## Highlights

• TLR3 overactivation induces robust type I interferon responses that suppress somatic cell nuclear reprogramming.
• Sustained activation of the IFNAR1-STAT1 signaling axis mediates the inhibitory effect of innate immune stress on reprogramming efficiency.
• Neutralizing IFN-β or blocking IFNAR1 restores nuclear reprogramming, without affecting cell proliferation or apoptosis.

## 1 Introduction

The nuclear reprogramming of somatic cells into pluripotency, a process of generating patient-specific cells from somatic cells, holds great promise for the field of regenerative medicine. The success of nuclear reprogramming to pluripotency intricately intertwines with innate immune responses. While a cell-autonomous innate immune response is indispensable for the nuclear reprogramming process [1], an excessive activation of the innate immune system, marked by an uncontrolled inflammatory response, has been shown to impede the efficacy of nuclear reprogramming processes [2].

Previous study suggests that overactivation of cell-autonomous innate immune signaling hinders nuclear reprogramming through decreased iNOS nuclear translocation, reduced MTA3 S-nitrosylation, heightened NuRD deacetylase activity, and diminished DNA accessibility [2]. However, given the indispensable role of innate immunity in nuclear reprogramming, inhibiting innate immune responses alone cannot serve as an effective strategy to restore nuclear reprogramming efficiency. Hence, exploring novel signaling targets of innate immune overactivation in nuclear reprogramming becomes imperative.

Innate immune activation is known to induce the release of large amounts of pro-inflammatory cytokines such as TNF-α and interferons (IFN). Intriguingly, B18R facilitates cellular reprogramming when mRNA-mediated OSKM gene delivery is used [3,4]. The B18R protein, an encoded protein from the vaccinia virus, binds to type I IFN and suppresses its antiviral response [5,6]. These findings imply that by mitigating the effects of IFN, B18R creates a more conducive environment for successful reprogramming. Another study indicates that IFN-γ, rather than IFN-β, impedes the transition to pluripotency [7].

Our objective is to explore and pinpoint the IFN signaling pathways responsible for the overactive state of innate immunity and its impact on impaired nuclear reprogramming. By manipulating the immune response during the reprogramming process, there is potential to create disease-specific or patient-specific cells more efficiently. This advancement could drive progress in personalized medicine and tissue regeneration. This modulation could pave the way for innovative developments in advanced regenerative medicine therapies and disease modeling techniques.

## 2 Methods

The datasets used and analyzed during the current study are available from the corresponding author on reasonable request. All methods are reported in accordance with ARRIVE guidelines.

### 2.1 Animal care and use

All animal studies were approved by the Institutional Animal Care and Use Committee of Guangzhou Laboratory. All experiments were performed in accordance with IACUC guidelines and regulations of Guangzhou Laboratory.

### 2.2 Chemicals and reagents

Alkaline Phosphatase (AP) staining kit was from Millipore. Anti-mouse Ifnar1 (clone MAR1-5A3) antibody, APC-Ifnar1 antibody and mouse IgG1 antibody was from Leinco Technologies. Hamster anti-mouse Ifn-β Antibody (clone MIB-5E9.1), hamster IgG antibody, mouse Ifn-β ELISA kit, mouse Ifn-γ ELISA kit and human Ifn-γ ELISA kit was from Biolegend. Human Ifn-β ELISA Kit and Click-iT™ EdU Alexa Fluor™ 488 Flow Cytometry Assay Kit was from life technology. HRP-conjugated anti-mouse or rabbit antibodies and anti-mouse Stat1 antibody was from Santa Cruz. Anti-mouse Lamin B1 antibody was from Cell Signaling. Taqman primers were from Life Technologies. FITC-Annexin V antibody, Annexin V binding buffer, and DAPI was from BD Biosciences. Polyinosinic polycytidylic acid (PolyI:C; PIC) was from Invivogen. Nuclear and Cytoplasmic Extraction Reagents were from Thermo fisher.

### 2.3 Cells

Doxycycline (dox)-inducible MEFs were isolated from embryos of transgenic R26rtTA; Col1a12lox-4F2A mice expressing the loxP-flanked, dox-inducible polycistronic 4F2A cassette [8] (Oct4, Sox2, Klf4, c-Myc; Jackson Laboratory). MEF cells were cultured and maintained in Dulbecco's modified Eagle's medium (Invitrogen) with 10 % fetal bovine serum and 1 % penicillin/streptomycin (5 % CO_2_, 37 °C) as previously described [9]. Passage 3 cells were used in all the experiments. Briefly, MEFs were seeded at 5 × 10^4^ per well in a six-well plate and were treated with dox 2 μg/ml every other day for a total of 12 days to induce reprogramming to pluripotency. Induced pluripotent stem cell (iPSC) colonies were examined at day 18. PIC 1000 ng/ml was treated every other day for the first 6 days. Neutralizing antibody and IgG control antibody, 2 μg/ml, were used every other day for the first 8 days.

### 2.4 Alkaline phosphatase staining

At day 18 of reprogramming, cells were fixed with 4 % paraformaldehyde for 1 min. Cells were then washed with TBST (20 mM Tris-HCl, pH 7.4, 0.15 M NaCl, 0.05 % Tween-20) twice. Alkaline phosphatase staining solution (prepared as recommended by the manufacturer) was used to incubate the cells for 15 min at room temperature prior to imaging.

### 2.5 RNA extraction and real-time PCR (RT-PCR)

mRNA was extracted from cultured cells using column purification (5 PRIME) and was reverse transcribed into cDNA by qScript cDNA SuperMix (Quanta Biosciences). Then Taqman primers targeting specific genes were used for RT-PCR and run on QuantStudio 3 real-time PCR system from Life Technologies. Gene expression data using the ΔΔCt method were normalized to the gene expression of the 18S control.

### 2.6 Western blot

Protein extraction was performed by lysing the cells in RIPA buffer supplemented with 1 × protease and phosphatase inhibitor cocktail. The total protein concentration was quantified using BCA assay (Pierce) and resolved on 4–12 % gradient SDS-polyacrylamide gels. The gel was transferred to a nitrocellulose membrane. The detailed immunoblotting procedure is described as previously [[10], [11], [12]]. Nuclear protein extract was collected using the Nuclear and Cytoplasmic Extraction Reagents.

### 2.7 Cell proliferation assay

Day 6 reprogramming MEF cells were examined for cell proliferation by EdU incorporation assay using Click-iT™ EdU Alexa Fluor™ 488 Flow Cytometry Assay Kit following the manufacturer's instructions.

### 2.8 Cell apoptosis assay

Day 6 reprogramming MEF cells were examined for apoptosis. Cells were stained with FITC-Annexin V in 1X Binding Buffer (0.01 M HEPES pH 7.4, 0.14 M NaCl, 2.5 mM CaCl_2_) at room temperature for 15 min. DAPI was used to stain dead cells.

### 2.9 Flow cytometry

Single cell suspension was stained with fixable live/dead dye to exclude dead cells. Cells were then stained with surface antigen staining and then with intracellular staining, such as BrdU. Samples were run on BD LSRII machine. Data were analyzed by FlowJo software.

### 2.10 ELISA assay

The ELISA assay was performed following the manufacturer's instructions.

### 2.11 Data analysis

Results were expressed as the mean ± SEM. Statistical comparisons between two groups were performed via Student's t-test. One-way ANOVA followed by multiple comparisons was used for statistical analysis of multiple groups. ∗*P* < 0.05 was considered significant.

## 3 Results

### 3.1 Overactivation of innate immunity increased type I IFN gene expression and secretion

**Fig. 1:**
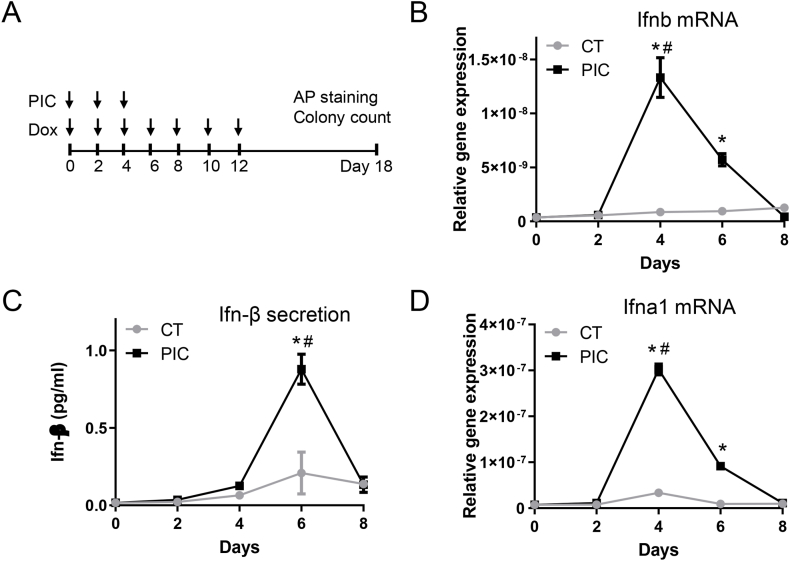
Overactivation of innate immunity increased type I interferon gene expression. In the CT group, dox-inducible OSKM MEFs were treated with dox every other day to initiate nuclear reprogramming. In the PIC group, cells received 1000 ng/mL PIC every other day for the first 6 days, in addition to dox treatment. A. Schematic representation of the experimental design. B. Ifnb gene expression at indicated time points. C. Measurement of Ifn-β protein level detected in culture medium collected at the specified time points. D. Ifna1 gene expression. All data are presented as mean ± S.E.M. ∗, P < 0.05 compared to the CT group at the same time point; #, P < 0.05 compared to the PIC group at day 0.

To investigate the impact of innate immune overactivation on type I IFN induction, we administered a high dosage of PIC to simulate TLR3 overactivation in dox-inducible OSKM MEFs. As illustrated in Fig. 1A, the CT group involved treating dox-inducible OSKM MEFs with dox every other day to initiate nuclear reprogramming. In contrast, the PIC group received 1000 ng/mL of PIC every other day for the first 6 days, concurrently with dox treatment. Cells were collected at the initial 8 days for the assessment of type I IFN gene expression.

In the CT group, the nuclear reprogramming process itself exhibited consistent and baseline levels of Ifnb mRNA over the initial 8 days, as depicted in Fig. 1B. Intriguingly, during the first 6 days of PIC treatment, a substantial surge in Ifnb gene expression was observed from day 2 to day 4, gradually declining to baseline by day 8. The ELISA-based detection of Ifn-β secretion in the collected culture medium at specified time points corresponded to the gene expression pattern shown in Fig. 1C. While the CT group showed a slight increase in Ifn-β secretion by day 6, PIC treatment notably elevated Ifn-β secretion at day 6, which subsequently returned to baseline by day 8.

Furthermore, we assessed Ifna1 gene expression, another component of the type I IFN family. Consistent with the pattern observed in Ifnb gene expression, Ifna1 mRNA levels also exhibited a sharp increase at day 4 following PIC treatment, aligning with the observed trend in Ifnb gene expression. These data highlight the pronounced impact of PIC-induced innate immune overactivation on the modulation of type I IFN gene expression and secretion during the initial phases of nuclear reprogramming.

### 3.2 PIC increased Ifnar1 surface expression and activated Ifnar1-Stat1 signaling

To further understand how the overactivation of innate immunity and induction of type I IFN might impede the efficiency of nuclear reprogramming, we assessed Ifnar1 surface expression and the downstream Ifnar1-Stat1 signaling. Ifnar1 is the primary receptor for type I IFNs [13].

**Fig. 2:**
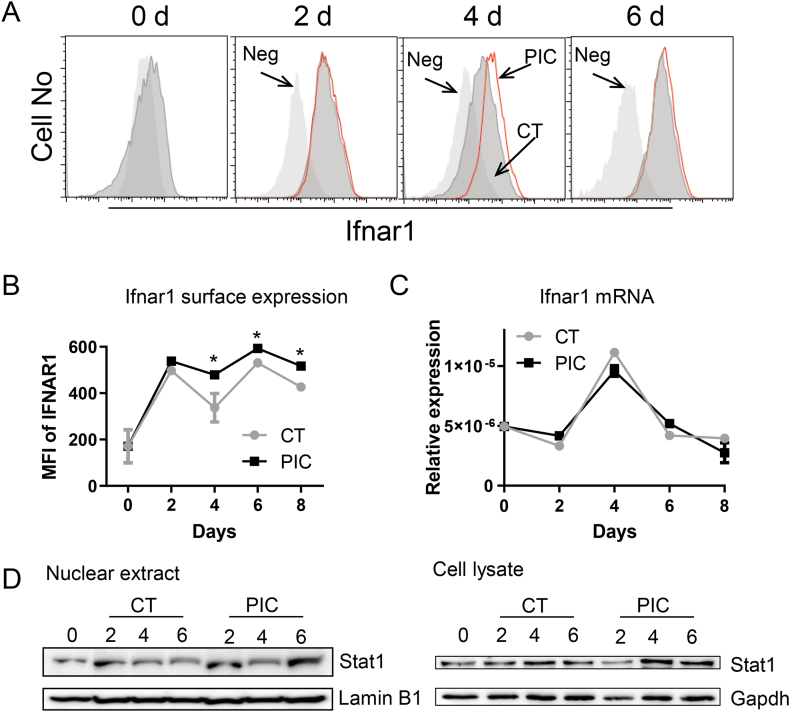
PIC increased Ifnar1 surface expression and activated Ifnar1-stat1 signaling. In the CT group, dox-inducible OSKM MEFs were treated with dox every other day to initiate nuclear reprogramming. In the PIC group, PIC at a concentration of 1000 ng/ml was administered every other day for the first 6 days in addition to dox treatment. A. Representative FACS histogram of Ifnar1 in MEFs collected at indicated time points. The red curve represents the PIC group. The dark shaded curve represents the CT group. The light shaded curve represents a negative staining control. B. Quantification of MFI of Ifnar1. C. Ifnar1 gene expression in MEFs treated with CT and PIC, collected at specified time points. D. Western blot analysis of Stat1 expression from nuclear and whole-cell lysates. All data are presented as mean ± S.E.M. ∗, P < 0.05 compared to the CT group.

Initially, we examined Ifnar1 cell surface expression in MEFs collected at designated time points using flow cytometry. In the CT group undergoing nuclear reprogramming alone, the Ifnar1 cell surface levels exhibited an increase at day 2, followed by slight fluctuations from day 2 to day 8, as depicted in Fig. 2A. The quantified mean fluorescence expression in Fig. 2B illustrates these observations. Interestingly, the PIC group showed a similar pattern of Ifnar1 cell surface expression as the CT group. However, from day 4 to day 8, the Ifnar1 cell surface levels were notably higher in the PIC group compared to the CT group. Additionally, we analyzed the Ifnar1 gene expression levels over the initial 8 days. Despite observing a sharp increase in Ifnar1 gene expression in both CT and PIC groups, there was no significant difference between the two groups (Fig. 2C).

Subsequently, we investigated the downstream Ifnar1-Stat1 signaling pathway by assessing the nuclear Stat1 levels via Western blot analysis. Notably, in the CT nuclear programming-only group, enhanced Stat1 levels were observed at day 2, coinciding with increased Ifnar1 cell surface expression at that time point, followed by a return to baseline levels by day 6. Conversely, in the PIC group, nuclear Stat1 levels were elevated at day 2 compared to the CT group. This elevation decreased at day 4 but continued to increase by day 6. The total Stat1 level remained stable at day 2 with or without PIC treatment (Fig. 2D).

These findings suggest that the nuclear reprogramming process itself led to increased Ifnar1 cell-surface expression, coupled with transient activation of Ifnar1-Stat1 signaling during the initial stages. However, PIC-induced overactivation further promotes Ifnar1 cell surface expression and induces prolonged activation of Ifnar1-Stat signaling. This cumulative effect emphasizes the role of innate immune overactivation in modulating Ifnar1 expression and signaling during nuclear reprogramming, potentially influencing its efficiency.

### 3.3 Blocking the Ifn-Ifnar1 axis restores PIC-impaired nuclear reprogramming

Upon observing enhanced type I IFN secretion, increased Ifnar1 cell surface expression, and intensified Ifnar1-Stat1 activation following PIC treatment, we hypothesize that Ifn-Ifnar1 axis activation is the major mediator of innate immune overactivation-impaired nuclear reprogramming. We then assessed whether the blockade of the Ifn-Ifnar1 axis could rectify PIC-induced impairments in nuclear reprogramming.

**Fig. 3:**
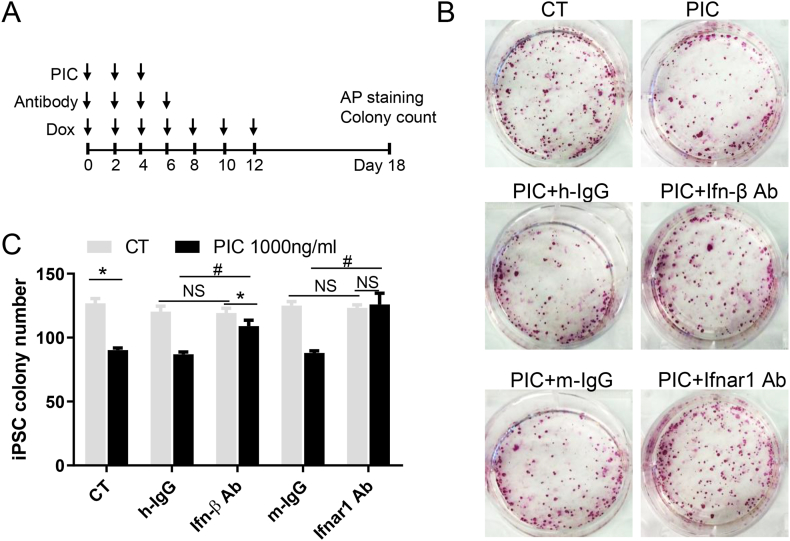
Antibodies blocking the Ifn-Ifnar1 axis restore PIC-impaired nuclear reprogramming. In the CT group, dox-inducible OSKM MEFs were treated with dox every other day to initiate nuclear reprogramming. In the PIC group, cells were treated with 1000 ng/ml PIC every other day for the first 6 days, in addition to dox treatment. In certain experimental groups, cells received treatment with Ifn-β neutralizing antibody or its control hamster IgG antibody, as well as Ifnar1 blocking antibody or its control mouse IgG antibody, every other day for the initial 8 days. A. Schematic representation of the experimental design. B. Representative images of iPSC colonies stained with AP (Alkaline Phosphatase). C. Quantification of iPSC colonies. All data are presented as mean ± S.E.M. ∗, P < 0.05 compared to the CT group; #, P < 0.05 compared to the IgG control-treated group.

We administered Ifn-β neutralizing antibody or control hamster IgG antibody, along with Ifnar1 blocking antibody or control mouse IgG antibody, every other day for the initial 8 days in conjunction with dox-only or dox-plus-PIC treatment (Fig. 3A).

Consistent with our prior finding [2], the overactivation of innate immunity hindered nuclear reprogramming efficiency, evident in the colony count by AP staining (Fig. 3B) and quantification (Fig. 3C). While Ifnar1 cell surface expression and Ifnar1-Stat signaling activation were observed in the nuclear programming-only group, the introduction of Ifn-β neutralizing antibody or Ifnar1 blocking antibody did not augment nuclear reprogramming efficiency beyond their respective control IgG groups, suggesting that Ifnar1-Stat signaling may not be a major barrier in nuclear reprogramming process itself.

Remarkably, the introduction of Ifnar1 blocking antibody completely reversed the impaired nuclear reprogramming efficiency induced by PIC treatment compared with its IgG control group. Similarly, Ifn-β neutralizing antibody substantially ameliorated the impaired nuclear reprogramming efficiency caused by PIC treatment compared with its IgG control group.

These observations suggest that the impairment of nuclear reprogramming due to innate immune overactivity occurs through the Ifn-Ifnar1 axis. While Ifn-β likely plays a major role in mediating this effect, other type I IFNs, such as Ifn-α, may also contribute to this phenomenon.

### 3.4 PIC treatment did not change the proliferation and apoptosis of OSKM-MEFs

**Fig. 4:**
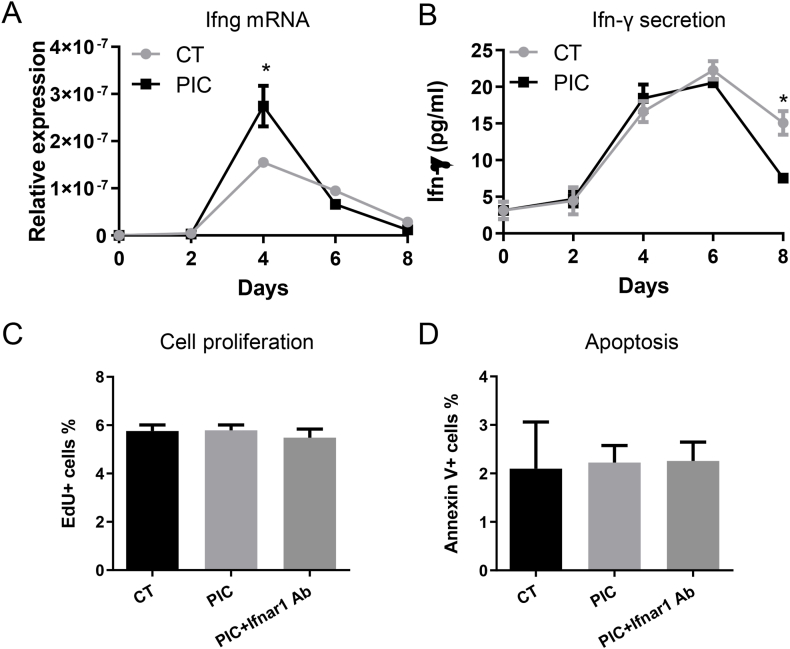
PIC treatment did not change the proliferation and apoptosis of OSKM-MEFs. In the CT group, dox-inducible OSKM MEFs were exposed to dox every other day to initiate nuclear reprogramming. In the PIC group, PIC was administered at a concentration of 1000 ng/mL every other day for the initial 6 days, in addition to dox treatment. In certain experimental groups, an Ifnar1 blocking antibody or its control, a mouse IgG antibody, was administered every other day for the first 8 days. A. Ifng gene expression. B. Levels of Ifn-γ detected in the culture medium using ELISA. C. Quantification of the percentage of EdU + cells. D. Quantification of the percentage of Annexin V+ cells. All data are presented as mean ± S.E.M. ∗, P < 0.05 compared to the CT group.

Since activation of innate immunity may induce both type I and type II IFN [14], we aimed to explore the involvement of type II IFN, such as Ifn-γ, in nuclear reprogramming. Although Ifn-γ is typically associated with T cell activation, our investigation during nuclear reprogramming revealed an increase in Ifng gene expression at day 4, returning to baseline levels by day 8. Notably, PIC treatment induced higher levels of Ifng gene expression compared to the CT group (Fig. 4A). However, while Ifn-γ secretion remained relatively similar between the CT and PIC groups within the initial 6 days, it notably decreased in the PIC group by day 8 compared to the CT group (Fig. 4B). These findings suggest that while the nuclear reprogramming process induces Ifn-γ secretion, PIC treatment did not augment it. These data suggest that the impairment of nuclear reprogramming by PIC might not be mediated by Ifn-γ.

Given that enhanced proliferation and reduced apoptosis are known to promote nuclear reprogramming efficiency, we sought to investigate whether PIC-induced impairment is associated with decreased proliferation or increased apoptosis. However, our data did not reveal any changes in cell proliferation, as determined by the EdU incorporation assay, or in apoptosis, assessed via Annexin V staining, following PIC treatment. Additionally, Ifnar1 blocking antibody did not alter cell proliferation or apoptosis rates (Fig. 4C and D). These observations suggest that the impaired nuclear reprogramming caused by PIC might not be directly linked to alterations in cell proliferation or apoptosis.

## 4 Discussion

The process of reprogramming cells into pluripotency, which involves generating patient-specific cells from somatic cells, represents a pivotal frontier in regenerative medicine. Strategies for generating iPSCs typically involve virus- or mRNA-based approaches that activate innate immunity. In clinical settings, inflammatory incidents experienced by numerous patients may reduce the efficiency of nuclear reprogramming in cells derived from them. Therefore, devising strategies to enhance nuclear reprogramming efficiency becomes imperative in such scenarios.

Innate immunity is the first line of defense against pathogens. Innate immune signaling pathways mediate the initial cellular response to damage or pathogens by releasing inflammatory cytokines, chemokines, and reactive oxygen species [15]. The potential role of cytokines and chemokines in nuclear reprogramming after innate immune overactivation remains largely unexplored. Type I IFN, expressed universally across cell types, plays a crucial role in the viral response. Intriguingly, B18R aids cellular reprogramming through RNA-mediated gene delivery [3,4]. This vaccinia virus-encoded protein binds to type I IFN and suppresses its antiviral response [5,6]. These reports suggest that by mitigating the effects of IFN, B18R fosters a more conducive environment for successful reprogramming. These studies indicate that type I IFN may play a critical role in nuclear reprogramming.

In this study, we aimed to examine whether the type I IFN-IFNAR1 axis plays a role in nuclear reprogramming with or without innate immune overactivation, and whether blocking this axis facilitates nuclear reprogramming. Interestingly, using dox-inducible OSKM MEFs as a nuclear reprogramming system, we found minimal baseline levels of type I IFN transcription and secretion. However, in the initial phase of nuclear reprogramming, cell surface Ifnar1 levels and nuclear Stat1 levels increased. Upon overactivation of innate immunity by high doses of PIC, a sharp increase in Ifn-β transcription and secretion occurred in the initial phase of nuclear reprogramming. Simultaneously, Ifnar1 cell surface levels were further increased, and nuclear Stat1 levels were enhanced compared to normal nuclear reprogramming conditions. Crucially, the Ifnar1 blocking antibody completely reversed the PIC-induced reduction in nuclear reprogramming efficiency, while the Ifn-β neutralizing antibody partially reversed the reduction, suggesting that other type I IFNs, such as Ifn-α, may also bind and activate IFNAR1 signaling.

Previous studies have suggested that optimal innate immunity induces alterations in the expression and functionality of epigenetic modifiers, enhancing DNA accessibility [1,16]. Heightened innate immune activation, however, leads to reduced NO generation and DNA accessibility, resulting in impaired nuclear reprogramming towards pluripotency. Overactivation of cell-autonomous innate immune signaling results in reduced iNOS nuclear translocation, decreased MTA3 S-nitrosylation, increased NuRD deacetylase activity, and consequently, reduced DNA accessibility [2].

While inhibiting iNOS might reverse impaired nuclear reprogramming caused by innate immune overactivation, this approach might impede the essential innate immune activation required for nuclear reprogramming. Directly inhibiting innate immunity may not be an optimal strategy to enhance nuclear reprogramming efficiency. Notably, the IFN-IFNAR1 axis appears to be a major downstream signaling pathway of innate immune activation that hinders nuclear reprogramming. We suspect that the IFN-IFNAR1 axis-mediated innate immune barrier might apply to other nuclear reprogramming processes, such as transdifferentiation or dedifferentiation. This cell-autonomous innate immune signaling could be evolutionarily conserved and applicable to cell fate switching.

Nuclear reprogramming to iPSCs holds great promise in regenerative medicine. Patient-specific iPSCs can, in principle, differentiate into cells of all three germ layers, enabling the understanding of disease mechanisms, use in cell therapy or regenerative medicine, or screening molecules for therapeutic strategies [17]. Overactivation of innate immunity is a major hindrance to nuclear reprogramming. Overactivation of innate immune signaling or chronic inflammation is, in general, detrimental to regenerative processes [[18], [19], [20], [21]]. For example, in the diabetic patient, chronic and persistent inflammation always manifests with a non-healing diabetic foot ulcer [22]. Here, we identified a major downstream signaling pathway that mediates the hindrance effect of innate immune overactivation in nuclear reprogramming. This study may have broad relevance to regenerative medicine.

## CRediT authorship contribution statement

**Zhimin Song:** Conceptualization, Data curation, Formal analysis, Investigation, Methodology, Project administration. **Yaofeng Wang:** Conceptualization, Data curation, Formal analysis, Investigation, Methodology, Project administration. **Yun Zhang:** Formal analysis, Investigation, Methodology. **Jingjing Chen:** Data curation, Formal analysis, Methodology. **Tinghong Zhang:** Data curation, Investigation, Methodology. **Shu Meng:** Conceptualization, Data curation, Formal analysis, Funding acquisition, Investigation, Methodology, Project administration, Resources, Software, Supervision, Validation, Visualization, Writing – original draft, Writing – review & editing.

## Declaration of competing interest

The authors declare no competing interests.

## Data Availability

Data will be made available on request.
